# Supplementary material for: Spatio-Temporal Variation of Groundwater Quality and Source Apportionment Using Multivariate Statistical Techniques for the Hutuo River Alluvial-Pluvial Fan, China
Source: Int J Environ Res Public Health. 2020 Feb 7;17(3):1055. doi: 10.3390/ijerph17031055 (PMC7036757; doi:10.3390/ijerph17031055)
Supplement: Supplementary file 1 [file ijerph-17-01055-s001.docx]

**Table S1.** Relative weight of physicochemical parameters and water quality standard.

| **Parameters** | **Water quality**  **standards** | **Weight(*W_i_*)** | **Relative weight(*RW_i_*)** |
| --- | --- | --- | --- |
| pH | 6.5–8.5 | 4 | 0.069 |
| EC | 500 | 4 | 0.069 |
| Na^+^ | 200 | 3 | 0.052 |
| Ca^2+^ | 75 | 3 | 0.052 |
| Mg^2+^ | 50 | 3 | 0.052 |
| Cl^-^ | 250 | 5 | 0.086 |
| SO_4_^2-^ | 250 | 5 | 0.086 |
| HCO_3_^-^ | 500 | 1 | 0.017 |
| NO_3_^-^ | 88.6 | 5 | 0.086 |
| NO_2_^-^ | 3.29 | 5 | 0.086 |
| TH | 450 | 4 | 0.069 |
| TDS | 1000 | 5 | 0.086 |
| COD | 3.0 | 5 | 0.086 |
| Fe | 0.3 | 3 | 0.052 |
| Mn | 0.1 | 3 | 0.052 |
| Sum |  | 58 | 1 |

Note: The EC, Mg^2+^, Ca^2+^ and HCO_3_^-^ refer to the World Health Organization (2011) standards, the other parameters refer to the grade III standard for groundwater quality in China (GB/T 14848-2017).

**Table S2.**Water quality classification ranged and types of water based on WQI values

| **Range** | **Type of ground water** |
| --- | --- |
| <50 | Excellent water |
| 50-100 | Good water |
| 100.1–200 | Poor water |
| 200.1–300 | Very poor water |
| >300 | Water unsuitable for drinking purposes |

**Figure S1.** Temporal variations of rainfall during 2015-2017.
